# Pharynx

**Published:** 1895-10-05

**Authors:** 


### Progress in Laryngology.

### LARYNGOLOGY.

### Pharynx.

**Latent Tuberculosis of the Three Tonsils.**—Professor Dieulafoy has recently read an important and interesting paper before the Paris Academy of Medicine.[1] He appears to have drawn his inspiration from some previous observations by Lermoyez, upon the presence of Koch's bacilli in adenoid vegetations with or without the co-existent naked eye appearance of tubercle, and conducted a series of experiments with a view to confirming them. Dieulafoy succeeded in developing general tuberculosis in eight out of sixty-one guinea pigs inoculated with fragments of enlarged faucial tonsil (equivalent to 13 per cent.), and in seven out of thirty-five animals inoculated with pieces of adenoid vegetations (20 per cent.). Care was taken to select the portions of tissue from persons in whom it was clear that the tonsilar affection was not secondary to pulmonary tuberculosis. Dieulafoy considers these patients to be suffering from a masked and sluggish type of tuberculosis of the pharynx, and that, aided by a natural predisposition to infection residing in the individual, the bacillus has gained access to the tonsils, palatine or pharyngeal, either by inhalation or by the food swallowed. These modes of approach have been proved possible by the investigations of Strauss and Chauveau respectively. The former proved the existence of virulent tubercle bacilli in the nasal cavities of healthy individuals frequenting rooms inhabited by consumptives, whilst the latter observed that animals fed on tuberculous food became infected through the lymphoid masses at the root of the tongue and isthmus of the fauces. The organism passes easily through the epithelial investment of the parts without any erosion of the surface being present. Inoculation is thought to be proved by the subsequent infiltration of the submaxillary and cervical glands. Dieulafoy distinguishes three stages of the disease: (1) That of latent infection of the tonsils and adenoids, with consequent increase of phagocytes and hypertrophy of the lymphoid tissue. (2) Inoculation of the glands, submaxillary and cervical. (3) Invasion of the lungs. Spreading takes place by the entrance of the bacillus into the lymphatic network associated with the tonsils, and its ultimate arrival into the venous system and right heart *via* the thoracic duct. The invasion of the glands appears to be hastened by an attack of some other infectious disease, such as scarlatina, whooping cough, or syphilis, suppuration taking place in them. But such a "generalisation" does not necessarily occur; phagocytosis may overcome the bacillary infiltration, the tonsils becoming hard and fibrosed, and a second stage evaded. The lungs, again, may repel the attack of the bacillus by setting up what Dieulafoy terms a "defensive hæmoptysis," the disease proceeding no further than the glands, and ending in recovery. Considerable time may elapse between the second and third stages. Believing that the mode of infection is chiefly by inhalation of an atmosphere, containing bacilli, the author recommends by way of prophylaxis, that the mouths and noses of children should be protected from the dust arising from tubercular sputa, and reminds us of the fact that certain foods, such as milk, cheese, and raw meat, are apt to contain and serve as vehicles for the bacillus. In order to increase the systemic powers of resistance, an abundant supply of fatty food should be given, and residence at the seaside is advisable The importance of Dieulafoy's communication is obvious, and it will be interesting to note the degree of confirmation which his experiments meet with at the hands of others. Cornil stated in the course of the discussion that out of seventy cases of adenoid hypertrophies which he had examined, he had only found evidence of tuberculous infiltration in four; whilst in enlarged faucial tonsils he had not once observed giant cells or other indications of tubercle. He also pointed out a source of fallacy in the experiments, viz., that the bacilli which had inoculated the guinea pigs need not necessarily have existed in the adenoid tissue itself, but might have been entangled in the mucous secretions contained in the crypts and surface irregularities of the organs from which the fragments were obtained.

**Giant Cell Systems in the Tonsil.**—Tuberculous disease of this body has, however, been observed by others. J. Purves Stewart[2] lately described such a case occurring in a child aged ten, who was the subject of chronic enlargement of the tonsils and cervical lymphatic glands, pharyngeal adenoids, and post scarlatinal otorrhœa. There was no history of tubercle. The tonsils had nothing abnormal in their external appearance, but scattered through their substance were large numbers of tuberculous giant cell systems, surrounded by the usual epithelioid and lymphoid cells. Tubercle bacilli were not discovered, nor was there any tuberculous tissue in the adenoids, but the enlarged cervical glands contained an abundance.

**The Role of the Tonsils in Infection of the Joints by Pyogenic Organisms.**—But phthisis pulmonalis is not the only constitutional disease, the origin of which has been traced to primary invasion of the tonsils. Some interesting observations have recently been made,[3] pointing to a similar association between enlarged tonsils and acute osteomyelitis. Kocher had believed the infective agents made their way to the bones and joints *via* the intestinal tract, but cases have occurred lately, in which comparatively insignificant tonsillar inflammations have been followed by osteomyelitis, suppurative periostitis, &c., &c., the micro-organisms in the bone affection being similar to those in the tonsil, *e.g.*, staphylococcus aureus and various streptococci. The following conclusions have been arrived at: (1) The tonsils may constitute a gate of entrance for pyogenic organisms, even in the absence of ulcers or diphtheritic lesions of the mucous membrane. (2) This rôle of the tonsils in the ætiology of osteomyelitis and various other suppurations is probably more important than that of the mucous membranes of the intestine and the respiratory tract. The inference from these observations is that cleanliness and care of the mouth and pharynx are of great importance as a means of preventing certain diseases, especially acute suppurating osteomyelitis.

[1] Bull. de l'Acad. de Méd., April 30, and May 7 and 14, 1895. [2] B.M.J., May 4, 1895. [3] Med. Week., Jan. 11, 1895.

